# Gastric Glomus Tumor (GGT) Mimicking Gastrointestinal Stromal Tumor (GIST): A Case Report and Literature Review

**DOI:** 10.7759/cureus.111814

**Published:** 2026-06-30

**Authors:** Ajith Vettuparambil, Mahesh S, Lekha V, Sajith K Mohan, John Mathew Manipadam, Ramesh Hariharan

**Affiliations:** 1 Gastrosurgery, Lakeshore Hospital and Research Centre, Cochin, IND

**Keywords:** gastric glomus tumor, gastric submucosal neoplasm, histopathology, immunohistochemistry, mesenchymal tumor

## Abstract

Gastric glomus tumor (GGT), a rare mesenchymal neoplasm, has only been reported in a few cases, and there are no established guidelines for its management. Diagnosing a gastric glomus tumor preoperatively is challenging due to its location and lack of specific clinical and endoscopic characteristics. Confirmation through biopsies before surgical resection is rare. Although most cases are benign, some may exhibit malignant features. Treatment typically involves wide local excision, with long-term follow-up necessary due to the potential for malignancy. Here, we present the case of a 41-year-old female who was diagnosed with a GGT postoperatively using histopathology and immunohistochemistry.

## Introduction

Gastric glomus tumors (GGTs) are uncommon mesenchymal neoplasms arising from modified smooth muscle cells of the glomus body, a structure involved in thermoregulation and blood flow control [1]. These tumors represent a mere 1% of all gastric mesenchymal tumors. First described in the mid-20th century, GGTs are predominantly benign and often present a diagnostic challenge due to their rarity and nonspecific symptoms [2].

A glomus tumor originates from the glomus body, an arterio-venous connection composed of tightly coiled capillaries surrounded by specialized muscle cells known as glomus cells [1]. In the gastrointestinal tract, GGT is commonly located in the stomach, particularly in the antrum. It typically emerges from the submucosal or muscular layers of the gastric wall, often without affecting the mucosal layer. GGT can protrude into the lumen or serosa of the stomach [1,3]. 

The preoperative diagnosis of GGT remains challenging because its clinical, endoscopic, and radiological features often overlap with those of other gastric subepithelial tumors. Gastrointestinal stromal tumors (GISTs) are the most common differential diagnosis, while leiomyoma, leiomyosarcoma, schwannoma, and neuroendocrine tumors should also be considered. Definitive diagnosis usually relies on histopathological examination and immunohistochemical analysis following surgical excision.

We present a case of a 41-year-old woman diagnosed with a GGT postoperatively, highlighting the diagnostic challenges and the importance of histopathological and immunohistochemical evaluation in establishing the correct diagnosis.

## Case presentation

A 41-year-old female presented with melena and abdominal discomfort, persisting for seven days. She had a history of left salpingectomy for tubal pregnancy six years ago and has undergone multiple abortions. On examination, her vital signs were normal, and abdominal and rectal examination were unremarkable.

Ultrasonography revealed a hypoechoic, hypervascular solid lesion measuring approximately 4.8 x 3.6 cm within the stomach. Contrast-enhanced CT scans revealed a submucosal, intensely vascular, endophytic polypoid lesion measuring 3.4 x 2.2 x 2.9 cm in the antropyloric region of the stomach, protruding into the pyloric canal (Figure 1). A feeding vessel originating from the left gastric artery (LGA) was observed adjacent to the lesion, indicative of a submucosal vascular gastric antral neoplasm.

**Figure 1:**
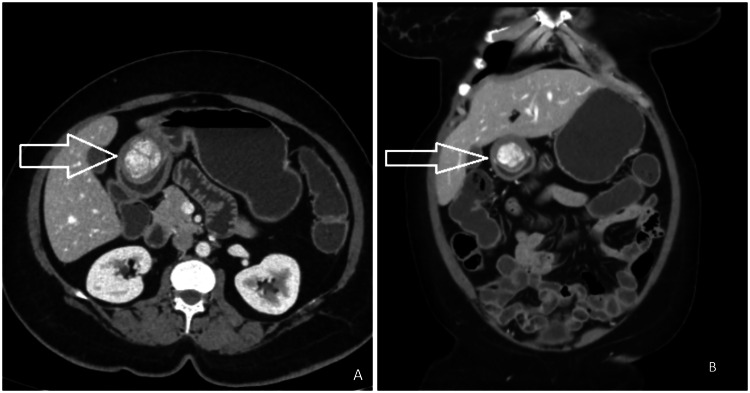
Contrast-enhanced computed tomography (CT) demonstrating a hypervascular gastric antral mass Panel A (axial view): Contrast-enhanced CT image demonstrating the gastric antral lesion (white arrow) with homogeneous enhancement, suggestive of a submucosal gastric neoplasm. Panel B (coronal view): A well-circumscribed, intensely enhancing submucosal mass (white arrow) arising from the anterior wall of the gastric antrum, projecting toward the pyloric canal, is seen.

Upper GI endoscopy identified a submucosal lesion measuring about 4 x 4 cm at the antrum, with central umbilication and a visible vessel noted at the central ulceration (Figure 2).

**Figure 2:**
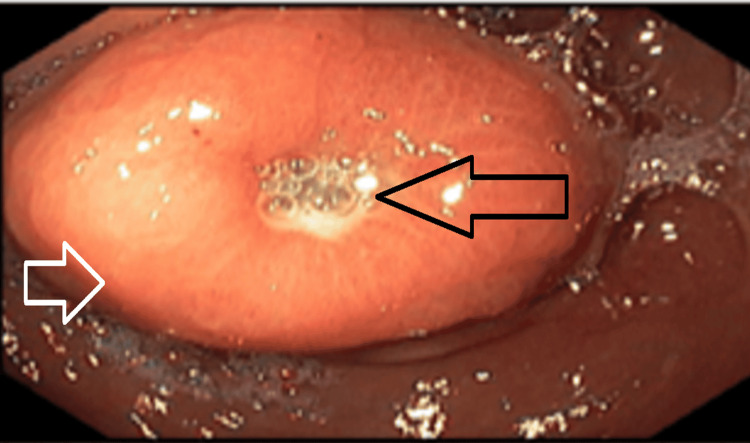
UGI endoscopy The white arrow points to the submucosal mass, and the black arrow points to the central umbilication. UGI: upper gastrointestinal

Based on its submucosal location, gastric antral origin, and imaging characteristics suggestive of a mesenchymal neoplasm, a preoperative diagnosis of GIST was made. Endoscopic ultrasound-guided fine-needle aspiration (EUS-FNA) was not attempted because of the high vascularity of the lesion on imaging. Blood investigations revealed a hemoglobin level of 9.3 g/dL (reference range: 12.0-15.0 g/dL), while all other laboratory parameters were within normal limits. The patient was subsequently taken up for laparoscopic wide local excision.

Laparoscopy revealed a 4x4cm tumor in the anterior wall of the gastric antrum with extension into the pylorus. She underwent laparoscopic excision of the tumor with an adequate margin. The postoperative period was uneventful.

Gross examination of the specimen showed a nodular mass covered with mucosa, measuring approximately 5x3.5x3.2 cm, exhibiting central umbilication measuring 0.8x0.7x0.2 cm (Figure 3). Upon sectioning, a well-circumscribed, gray-white, hemorrhagic, lobulated, and firm mass measuring 4.7x2.4x2.4 cm was observed in the muscle plane, extending into the umbilicated area of the mucosal aspect (Figure 4).

**Figure 3:**
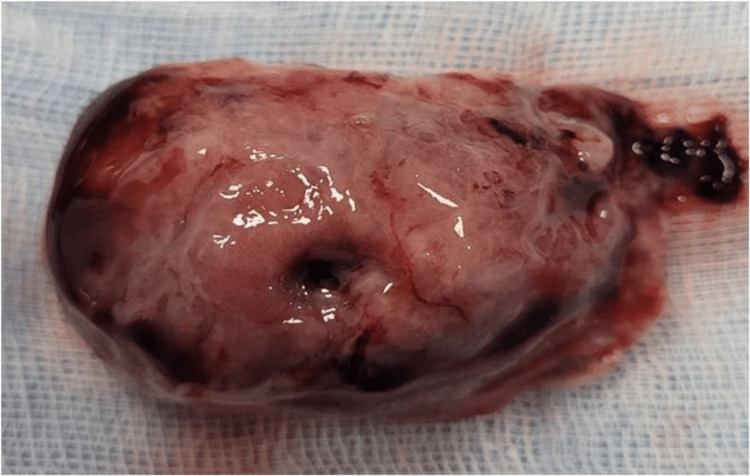
Macroscopic gross specimen; laparoscopic wide local excision specimen

**Figure 4:**
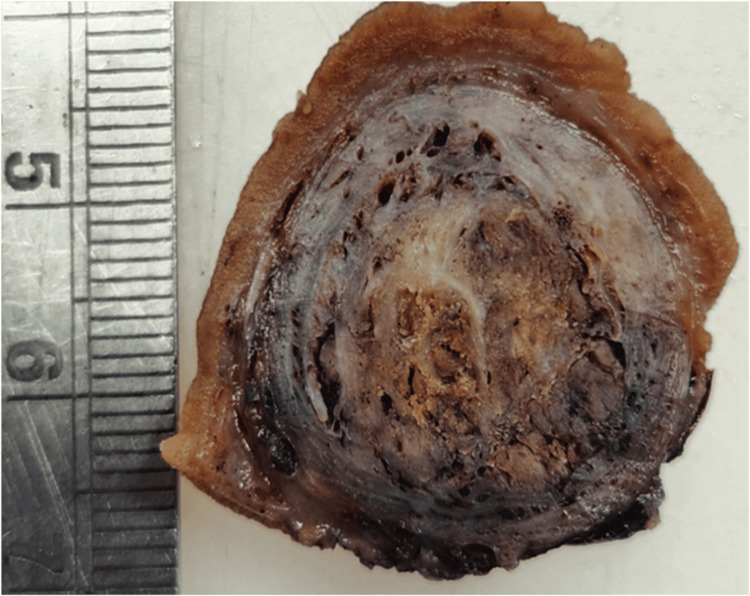
Cut section of the wide local excision specimen

Microscopic examination revealed a neoplasm in the muscularis propria, comprising dilated vascular spaces surrounded by nests, trabeculae, and cords of neoplastic cells. These cells are projected into the vascular spaces as polypoid projections. The neoplastic cells exhibited predominantly uniform features, with a moderate amount of clear cytoplasm, round nuclei with granular chromatin, mild nuclear enlargement, and focal elongation. Mitotic figures were observed at a rate of 0-2/10 mm2. The intervening stroma appeared hyalinized. The neoplasm infiltrated into the mucosa, causing ulceration. The deep margin showed a clearance of 0.4 cm, while the tumor did not involve the mucosal margin. Lymphovascular invasion was present, and no perineural invasion was observed (Figure 5).

**Figure 5:**
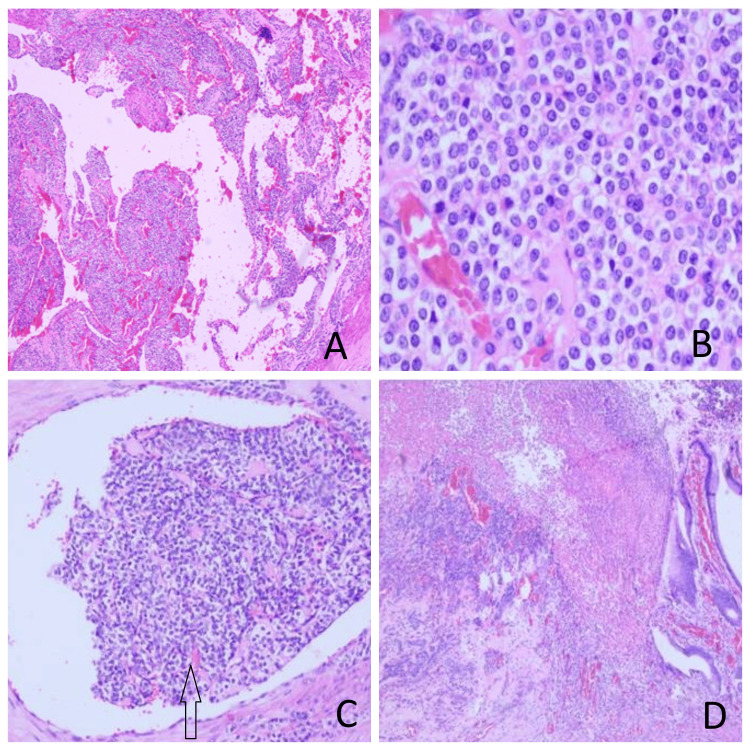
Histopathology: microscopic findings A. Dilated vascular spaces surrounded by nests of neoplastic cells (magnification x40), B. Uniform cells with clear cytoplasm and defined cell borders (magnification x400), C. Lymphovascular emboli (black arrow) (magnification x100), D. Ulceration extending into the overlying gastric mucosa (magnification x40).

On immunohistochemistry (IHC) evaluation, neoplastic cells exhibited diffuse positivity for vimentin and caldesmon and patchy positivity for SMA (smooth muscle actin). The Ki-67 index was 2-4%. The cells were negative for CK (cytokeratin) and chromogranin. CD-31 highlighted admixed vascular spaces. Both the histopathology and IHC features were consistent with glomangioma (GGT) (Figure 6).

**Figure 6:**
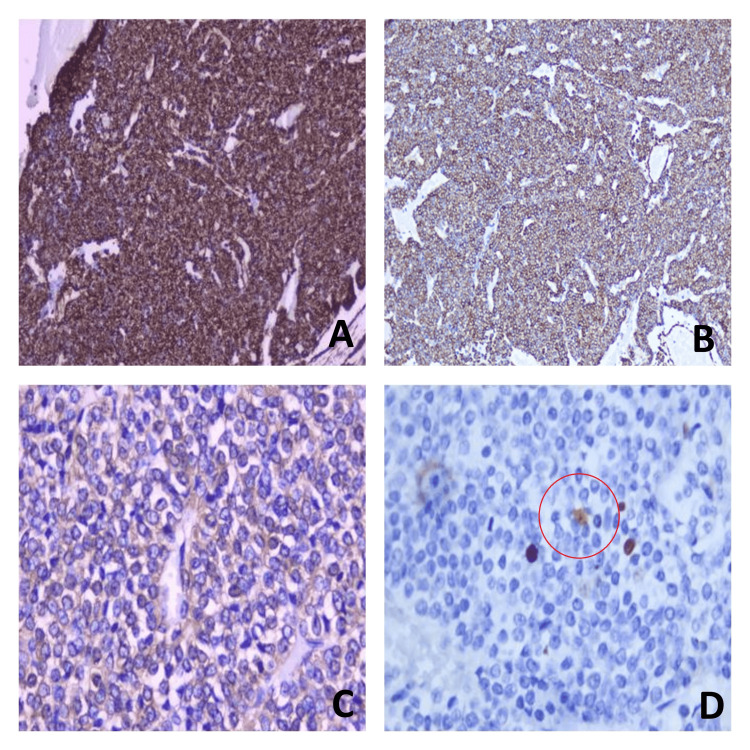
Immunohistochemistry findings (A) Diffuse cytoplasmic positivity for vimentin, (B) Diffuse positivity for caldesmon, (C) Patchy positivity for smooth muscle actin (SMA). (D) Ki-67 proliferation index of approximately 2-4% (marked region). (Magnification: A & B X100, C & D X400)

After discharge, the patient was followed up regularly in the outpatient department every three to six months. Approximately 24 months after surgery, she remains asymptomatic, with no clinical or radiological evidence of recurrence.

## Discussion

The GGT presents diagnostic challenges due to its similarity to other gastric submucosal lesions, often misdiagnosed as GISTs. Patients may be asymptomatic initially but may later experience symptoms such as epigastric pain, nausea, vomiting, fatigue, melena, and occasional upper gastrointestinal bleeding [4,5].

GGT diagnosis depends on examining surgical specimens pathologically and immunohistochemically. Biopsies from endoscopic ultrasound (EUS) or upper gastrointestinal endoscopy are rarely used for diagnosis. The main diagnostic methods for evaluating GGT are endoscopic ultrasound (EUS) and computerized tomography (CT) [6]. EUS is advantageous for identifying the layer of tumor origin, while CT is better for depicting its characteristics. The CT scan of the abdomen may suggest a well-circumscribed submucosal mass in the stomach, characterized by homogeneous density on unenhanced images. This mass exhibits substantial enhancement on arterial phase images and persistent enhancement on portal venous phase images [6,7].

Due to its intramural location, diagnosing GGT by conventional endoscopic biopsy is often challenging. Consequently, the diagnosis is usually established following histopathological examination of the surgical specimen, with immunohistochemistry playing a pivotal role in confirming the diagnosis [8]. Although immunohistochemical findings aid in tumor characterization, they do not reliably predict biological behaviour, and the potential for metastasis cannot be ruled out. Folpe et al. proposed criteria for assessing malignant potential based on deep location, size (>2 cm), atypical mitotic figures, moderate-to-high nuclear grade, and increased mitotic activity (>5/50 high-power fields) [9]. In the present case, despite the tumor measuring >2 cm and demonstrating lymphovascular invasion, the low mitotic rate (0-2/10 mm²), low Ki-67 index (2-4%), and absence of significant nuclear atypia support its classification as a benign glomus tumor. However, the presence of lymphovascular invasion warrants close long-term follow-up. Reported metastatic sites of malignant glomus tumors include the brain, bones, small intestine, lungs, and liver [9].

Regarding surgical treatment, the preferred option is wide local excision with clear margins [10]. Extended margins of resection or extensive lymph node removal are not necessary. Additionally, the excision of a GGT via EUS has been suggested [10]. However, GGTs contain numerous blood vessels, increasing the risk of intraoperative bleeding. Due to the potential for malignancy, long-term follow-up is recommended.

## Conclusions

Gastric glomus tumors (GGTs) are rare mesenchymal neoplasms that are typically benign but frequently mimic other gastric neoplasms, particularly gastrointestinal stromal tumors (GISTs), in the preoperative setting. Definitive diagnosis relies on histopathological examination and immunohistochemistry, while complete surgical excision remains the treatment of choice with good outcomes. Although most GGTs behave in a benign manner, occasional cases of metastasis have been reported. Therefore, long-term follow-up is recommended, particularly in patients with features suggestive of malignant potential.
